# Efficacy of Massage Therapy for Symptom Management in Cancer Patients: A Systematic Review and Meta-Analysis

**DOI:** 10.3390/healthcare13243268

**Published:** 2025-12-12

**Authors:** Arda Uzunoglu, Paula Matta-Diaz, Valeria Bustos-Gajardo, Javiera Obreque-González, Gloria Cifuentes-Suazo, Guinevere Granite, Mathias Orellana Donoso, Pablo Nova Baeza, Gustavo Oyanedel-Amaro, Alvaro Becerra Farfan, Juan Sanchis-Gimeno, Juan José Valenzuela-Fuenzalida, Jessica Paola Loaiza Giraldo, Jose E. León-Rojas

**Affiliations:** 1 Republic of Turkey Ministry of National Education, Kocaeli 06420, Turkey; arda-uzunoglu@hotmail.com; 2 Departamento de Morfología, Facultad de Medicina, Universidad Andrés Bello, Santiago 8370146, Chile; pau.matta2640@gmail.com (P.M.-D.); valex.bustos@gmail.com (V.B.-G.); javi244199@gmail.com (J.O.-G.); mathor94@gmail.com (M.O.D.); pablo.nova@usach.cl (P.N.B.); juan.kine.2015@gmail.com (J.J.V.-F.); 3 Facultad de Medicina, Carrera de Odontología, Universidad Católica de la Santísima Concepción, Av. Alonso de Ribera 2850, Concepción 4090541, Chile; gbcifuentess@gmail.com; 4 Department of Surgery, F. Edward Hébert School of Medicine, Uniformed Services University of the Health Sciences, Bethesda, MD 20814, USA; guinevere.granite@usuhs.edu; 5 Escuela de Medicina, Universidad Finis Terrae, Santiago 7501015, Chile; 6 Facultad de Ciencias de la Salud, Universidad Autónoma de Chile, Santiago 7500912, Chile; g.oyanedelamaro@gmail.com; 7 Departamento de Ciencias Química y Biológicas, Facultad de Ciencias de la Salud, Universidad Bernardo O’Higgins, Santiago 8370993, Chile; alvaro.becerra@ubo.cl; 8 GIAVAL Research Group, Department of Anatomy and Human Embryology, Faculty of Medicine, University of Valencia, 46001 Valencia, Spain; juan.sanchis@uv.es; 9 Faculty of Health and Social Sciences, Universidad de Las Américas, Santiago 8370040, Chile; 10 Facultad de Ciencias de la Salud, Universidad Central del Valle del Cauca (UCEVA), Tuluá 763022, Colombia; yessica.loaiza01@uceva.edu.co; 11 Cerebro, Emoción y Conducta (CEC) Research Group, Escuela de Medicina, Universidad de las Americas (UDLA), Quito 170124, Ecuador

**Keywords:** massage therapy, reflexology, cancer, complementary medicine, supportive care, symptom management, relaxation therapy

## Abstract

**Background:** Cancer and its treatments frequently lead to physical and psychological symptoms that negatively affect quality of life. Massage therapy has been proposed as a complementary intervention to reduce symptom burden through its effects on stress regulation and autonomic balance. This systematic review and meta-analysis evaluated the effectiveness of massage therapy in patients with cancer. **Methods:** A systematic search was conducted in MEDLINE, Web of Science, Scopus, Embase, Google Scholar, and CINAHL. Search terms included “massage therapy,” “reflexology,” “massage,” and “cancer.” Randomized controlled trials comparing massage therapy with placebo or standard care and reporting quantitative outcomes were eligible. Seven studies met inclusion criteria for the meta-analysis. **Results:** Compared with control conditions, massage therapy was associated with significant improvements in several outcomes: Behavioral Symptoms Frequency (BSF) (MD = −12.54; 95% CI: −18.70 to −6.38; *p* < 0.0001), Quality of Life Questionnaire (QLQ) scores (SMD = 10.10; 95% CI: 1.21 to 19.00; *p* = 0.03), Spielberger State–Trait Anxiety Inventory (STAI) scores (MD = −3.97; 95% CI: −4.63 to −3.31; *p* = 0.0001), and Visual Analog Scale (VAS) symptom intensity (MD = −1.09; 95% CI: −2.11 to −0.07; *p* = 0.04). Overall certainty of evidence was limited by methodological heterogeneity and risk of bias. **Conclusions:** Massage therapy may provide short-term improvements in selected physical and psychological symptoms in cancer patients and may serve as a supportive complementary intervention. However, the evidence remains limited, and well-designed trials with standardized protocols are needed to strengthen the reliability of these findings.

## 1. Introduction

Cancer is a complex, multifactorial disease arising from the interaction of genetic mutations, epigenetic alterations, disruptions in cellular communication, and changes in the tumor microenvironment. Rather than originating from a single cause, carcinogenesis results from an interplay of molecular, environmental, and immunological factors that progressively lead to abnormal cell differentiation and uncontrolled proliferation. It represents a major public health problem due to its high morbidity and mortality. Global cancer incidence continues to increase, largely due to demographic expansion, population aging, and improved diagnostic capacity, although some cancers have also risen for multifactorial, non-demographic reasons [1]. The most common cancers worldwide include breast, lung, and colorectal malignancies [2]. According to WHO (2024), cancer incidence is expected to increase by 77% by 2050 compared with 2022, mainly as a result of population aging and changes in exposure to risk factors [2]. Cancer treatment is interdisciplinary and may pursue curative, palliative, or life-prolonging goals, typically through surgery, chemotherapy, radiotherapy, or biological therapies. While these treatments aim to destroy malignant cells, they also generate collateral damage to healthy tissues, leading to acute and chronic complications such as mucositis, dermatitis, alopecia, myelosuppression, neuropathies, infertility, and symptoms such as pain, fatigue, nausea/vomiting, anxiety, and emotional distress [1,3].

In recent years, complementary and alternative therapies have gained relevance in oncology as supportive measures to improve quality of life and mitigate treatment-related symptoms. Massage therapy one of the most widely used complementary interventions has shown potential benefits for symptom relief through modulation of physiological and psychological pathways [4]. Massage stimulates cutaneous and deep mechanoreceptors that transmit signals to the central nervous system and neuroendocrine pathways, contributing to reduced cortisol and catecholamine release, increased parasympathetic activity, improved lymphatic and blood flow, and decreased anxiety and stress [5,6]. Additionally, some evidence suggests that massage may enhance immune function by increasing Natural Killer cell activity [6,7].

Although other complementary techniques such as meditation or acupuncture have been explored in supportive cancer care, they are not the focus of this review and will only be mentioned when relevant to contextualize the current evidence.

Massage therapy involves the application of rhythmic compressions with varying pressure and speed to induce relaxation and therapeutic effects. From a physiological perspective, massage can increase local temperature, enhance circulation, promote lymphatic drainage, and trigger reflex responses mediated by the autonomic nervous system [8,9]. These mechanisms have also been described in physiological studies of manual therapy, which report decreases in cortisol and catecholamines, parasympathetic activation, and improved hemodynamic responses [10].

Despite growing interest, evidence on the efficacy of massage therapy in oncology remains fragmented across heterogeneous studies. Therefore, continued investigation is required to clarify its therapeutic contribution to symptom control. Thus, the aim of this systematic review and meta-analysis is to evaluate the efficacy of body massage as a complementary therapy for symptom reduction and quality-of-life improvement in cancer patients. This review focuses exclusively on massage interventions compared with control conditions and does not assess meditation or other complementary modalities unless they appear as co-interventions in the primary studies.

## 2. Methods

### 2.1. Protocol and Registration

This systematic review and meta-analysis was conducted and reported in accordance with the Preferred Reporting Items for Systematic Reviews and Meta-Analyses (PRISMA) statement [11]. The protocol was registered with the Open Science Framework (OSF) and is available at: https://osf.io/e2u46 (accessed on 2 December 2025)

### 2.2. Literature Search

We systematically searched the following electronic databases: MEDLINE (via PubMed), EMBASE, Scopus, the Cochrane Central Register of Controlled Trials (CENTRAL), the Cumulative Index to Nursing and Allied Health Literature (CINAHL), and Web of Science. Searches covered records from inception to May 2025. We included randomized or controlled clinical trials published in English or Spanish.

The search strategy followed a PIO framework, where the Population was cancer patients, the Intervention was massage therapy, and the Outcomes were symptom reduction and quality-of-life improvement. The search strategy was developed using Medical Subject Headings (MeSH), Emtree terms, and free-text keywords. Controlled vocabulary and synonyms were adapted for each database. The core search terms included combinations of: “massage,” “massage therapy,” “therapeutic massage,” “cancer,” “neoplasms,” “oncology patients,” “symptom relief,” and “quality of life.” Irrelevant terms such as acupuncture, reflexology, and musculoskeletal manipulations were intentionally excluded to maintain alignment with the study objective.

Two authors (J.J.V.-F. and J.O.-G.) independently screened titles and abstracts retrieved from the searches. Full texts were obtained for all potentially eligible studies. A third reviewer (P.M.-D.) resolved any disagreements. No automated tools were used during the screening process.

### 2.3. Study Selection. Eligibility Criteria and Study Selection Process

The study selection process was conducted in two stages: first as a systematic review, followed by a meta-analysis of studies that met the requirements for quantitative synthesis. Eligibility criteria were defined using a PIO framework (Population, Intervention, Outcome) to ensure alignment with the study objective.

Population: Adults or adolescents diagnosed with any form of cancer and experiencing cancer-related symptoms such as pain, anxiety, fatigue, or mood disturbances. Intervention: Any type of massage therapy, including body massage, Swedish massage, therapeutic massage, or other manual massage techniques administered by trained personnel. Outcomes: Symptom reduction (e.g., pain, anxiety, fatigue), mood improvement, or enhanced quality of life measured with validated scales. Study Designs Included: Randomized controlled trials (RCTs) and controlled clinical or experimental studies were included in the quantitative meta-analysis. Qualitative or mixed-methods studies were included only in the narrative portion of the systematic review and were not incorporated into the meta-analysis.

Exclusion Criteria Studies were excluded if they were case reports, case series, editorials, letters, reviews, or non-human studies; lacked a control or comparison group; evaluated complementary therapies other than massage (e.g., acupuncture, reflexology, aromatherapy without massage); or included participants without cancer. Two reviewers independently screened titles and abstracts, followed by full-text assessment of potentially eligible studies. Disagreements were resolved through discussion or consultation with a third reviewer.

### 2.4. Data Extraction and Quality Assessment

Two reviewers (J.L.-R. and P.N.-B.) independently extracted data from all included studies using a predefined data extraction form developed specifically for this review. The tool was piloted on three studies to ensure clarity and consistency before full data extraction was performed. Extracted variables were based on the PIO framework and included: study characteristics (authors, year, country, study design, sample size, and participant demographics), details of the intervention (type of massage, intervention duration and frequency, and provider characteristics), comparator type, and reported outcomes related to symptom reduction, mood, and quality of life. We also extracted statistical results necessary for quantitative synthesis [12,13,14,15].

The methodological quality of included studies was assessed independently by the same two reviewers using the Cochrane Risk of Bias (RoB1) tool [16]. This tool evaluates seven domains: random sequence generation, allocation concealment, blinding of participants and personnel, blinding of outcome assessment, incomplete outcome data, selective outcome reporting, and other sources of bias. Each domain was rated as “low,” “unclear,” or “high” risk of bias. Disagreements were resolved through discussion or, when necessary, by a third reviewer (J.J.V.-F.). Inter-rater reliability was assessed using Cohen’s kappa statistic, which indicated substantial agreement (κ = 0.88).

### 2.5. Data Synthesis and Analysis of Scales

For all included studies, outcome data were extracted from validated scales commonly used in oncology research, such as the HADS, STAI, EORTC QLQ-C30, VAS, and other symptom-related instruments. For each scale, we used the reported mean and standard deviation at baseline and post-intervention (or change scores when available) to calculate standardized mean differences (SMDs). Higher scores were interpreted according to each scale’s directionality, and values were standardized so that negative SMDs consistently indicated symptom improvement when appropriate.

Effect sizes were calculated using Cohen’s d and interpreted as trivial (<0.20), small (0.20–0.49), moderate (0.50–0.79), or large (≥0.80). Pooled SMDs were estimated using either the Hartung–Knapp–Sidik–Jonkman random-effects method when heterogeneity was present or the Mantel–Haenszel fixed-effect model when heterogeneity was low. The Hartung–Knapp–Sidik–Jonkman approach provides more robust variance estimation in random-effects meta-analyses, particularly in the presence of a small number of studies.

Heterogeneity was assessed using the I^2^ statistic, interpreted as follows: 0–40% (not important), 30–60% (moderate), 50–90% (substantial), and 75–100% (considerable) heterogeneity. Forest plots were inspected to evaluate consistency of effect directions and overlap of confidence intervals. All meta-analyses were conducted using RevMan 5.4, and results are reported as SMDs with their corresponding 95% confidence intervals.

### 2.6. Rating the Quality of Evidence

The certainty of the evidence for each outcome was assessed using the Grading of Recommendations, Assessment, Development, and Evaluation (GRADE) approach. Evidence quality was classified as high, moderate, low, or very low according to GRADE criteria [17]. We used GRADEpro to import effect estimates from RevMan 5.4 and generate the Summary of Findings table, which is available in Supplementary Table S3 (http://links.lww.com/MD/K340, accessed on 2 December 2025).

## 3. Results

### 3.1. Study Selection

**Figure 1:**
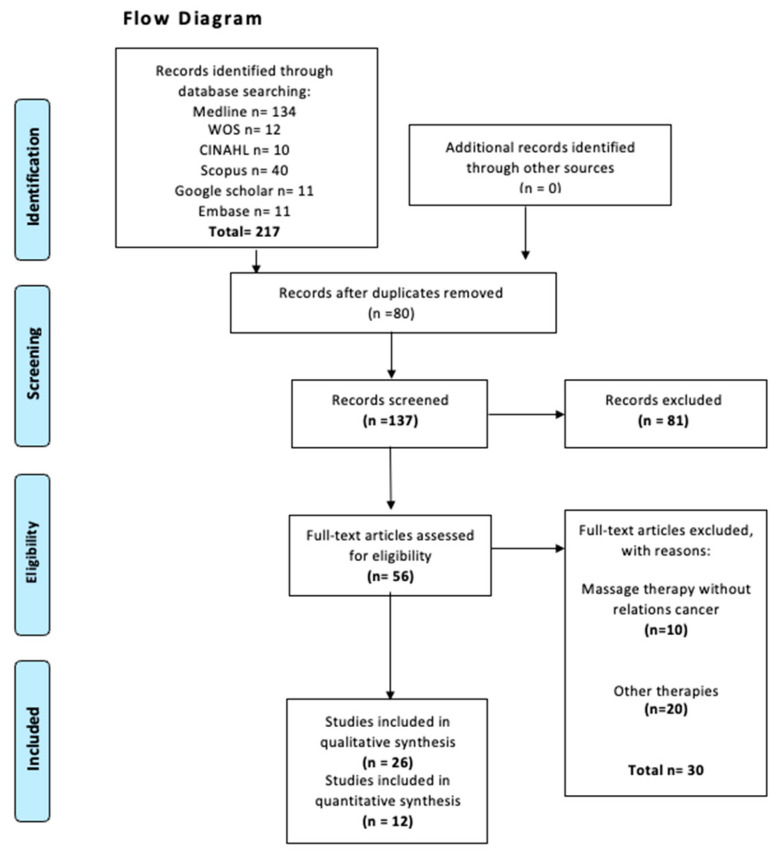
Flow diagram search.

The electronic search identified 217 records. After removing duplicates and screening titles and abstracts, 26 studies were selected for full-text review. Of these, 26 studies met the criteria for inclusion in the qualitative synthesis, and 12 randomized controlled trials fulfilled the additional requirements for inclusion in the quantitative meta-analysis. The study selection process is detailed in the PRISMA flow diagram (Figure 1). No additional eligible studies were identified through clinical trial registries. A list of excluded full-text studies with reasons for exclusion is provided in Supplementary Table S1.

### 3.2. Study Characteristics

Table 1 provides a summary of all studies included in the qualitative synthesis. Twelve studies were eligible for the comparative analysis and evaluated different types and durations of massage therapy in patients undergoing cancer treatment [5,6,7,18,19,20,21,22,23,24,25,26]. These studies were published between 2004 and 2022 and were conducted in the United States, Sweden, Iran, Canada, Germany, and Japan. Several studies were multicenter, increasing the geographic diversity of the sample.

A total of 1019 patients were included across the 12 studies, with 555 in the experimental (massage) group and 464 in the control group. The mean ages were 55.92 (±6.26) years in the experimental group and 55.84 (±9.02) years in the control group. Follow-up duration averaged 5.43 weeks, ranging from 24 h to 6 months. For the meta-analysis, seven studies [5,6,7,18,19,20,21] were included. The remaining studies could not be pooled due to differences in outcomes or follow-up duration. The seven meta-analyzed studies included a total of 318 participants, with mean ages of 53.46 (±6.60) and 53.28 (±11.10) years for the experimental and control groups, respectively.

### 3.3. Risk of Bias Assessment in Individual Studies

**Figure 2:**
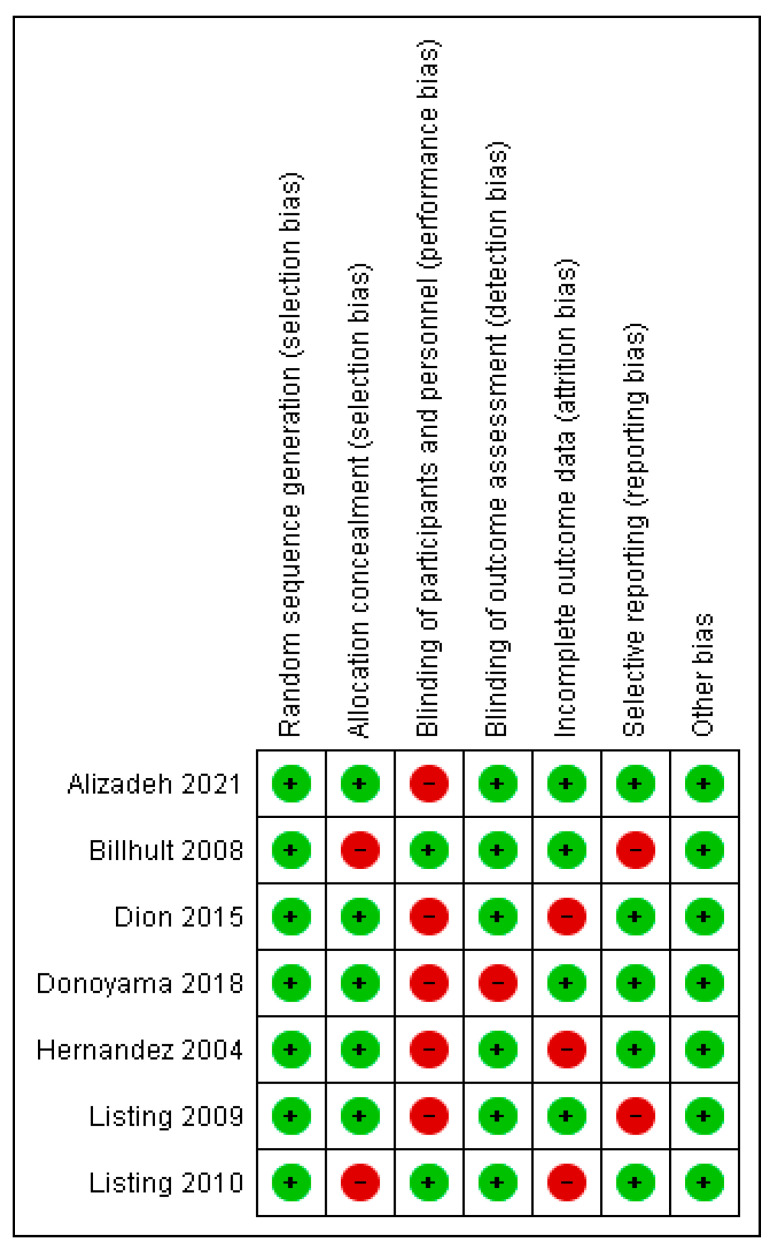
Risk of bias assessment in individual studies included in the review. Green (+) indicates low risk of bias; red (−) indicates high risk of bias [6,7,18,23,24,25,27].

**Figure 3:**
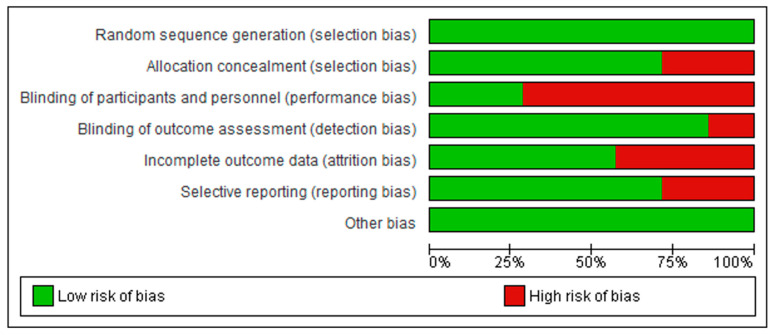
Summary of the risk of bias assessment across the included studies, showing the proportion of studies rated as low or high risk for each methodological domain.

The RoB2 assessment is presented in Figure 2 and Figure 3. For random sequence generation, 100% of the studies were classified as having a “low risk” [5,6,7,18,23,24,25]. In allocation concealment, 71% were classified as having a “low risk” of bias [5,7,18,24,25], whereas 29% presented a “high risk” or “unclear risk” [6,23]. For blinding of participants and personnel, 28.6% of the trials received a “low risk” of bias rating [6,23], while 71.4% were judged as having a “high risk” [5,7,18,24,25]. For the blinding of outcome assessment, 71% of the trials were scored as “low risk” [5,6,7,18,23,24], and 29% as “high risk” [25]. For incomplete outcome data, 71% received a “low risk” rating [5,6,7,24,25], while 29% showed a “high risk” [18,19]. Finally, for the selection of reported results, 57% of the trials were scored as “low risk” [5,7,18,23,25], and 43% were scored as having a “high” risk of bias [6,24].

#### 3.3.1. Dependent Evaluative Effect

Of the included studies, eight of them [5,6,7,18,19,21,22,25] reported no dependent evaluative effect, while the remaining studies [14,23,24,26] reported such a relationship. These studies mention that the personal characteristics of the therapist, interpersonal interactions and communication, and the recipient’s expectation of a positive outcome influence the effect of massage on the observed mood disorders.

#### 3.3.2. Types of Cancer of the Included Subjects

The cancers reported by the studies were varied, with the following systems being identified. For digestive system cancer, this was reported in the work of Alizadeh et al. [27], Kutner et al. [20], López et al. [21], and Toth et al. [22]. Addressing patients whose cancer affects the reproductive system, we find the studies of Donoyama et al. (2018) and Toth et al. (2013) [22,25].

Regarding the integumentary system, specifically breast cancer, the following works were reported: López et al. (2022), Hernandez-Reif et al. (2004), Listing et al. (2009), Listing et al. (2010), Dion et al. (2016), Kutner et al. (2008), Toth et al. (2013), and Billhult et al. (2008) [6,7,18,20,21,22,23,24]. Finally, both the study conducted by Campeau et al. (2007) and Kutner et al. (2008) do not specify the type of cancer present in their patients. However, the first author mentioned that patients underwent radiotherapy treatment, and the second author indicated that the patients had advanced cancer in palliative care [20,26]. Similarly, in the work of Post-White et al. (2009), which focuses on pediatric cancer patients, the exact type of cancer was also not specified [28].

The studies conducted by Hernandez-Reif et al. (2004), Listing et al. (2009), and Listing et al. (2010) report that the patients in their research are in stage I and II of breast cancer [7,23,24]. On the other hand, López et al. (2022) works with patients whose cancer is classified as stage I, II, III, and IV [21]. Additionally, in the research of Kutner et al. (2008), Kutner et al. (2010), and Toth et al. (2013), the treated patients are in a more advanced stage where metastasis is already present [20,22,29]. Finally, in the works of Alizadeh et al. (2021), Donoyama et al. (2018), Campeau et al. (2007), Post-White et al. (2009), Dion et al. (2016), and Billhult et al. (2008), the stage of cancer in the patients is not specified [6,18,25,26,27,28].

### 3.4. Synthesis of Results

#### 3.4.1. Forest Plot BSF Scale

**Figure 4:**
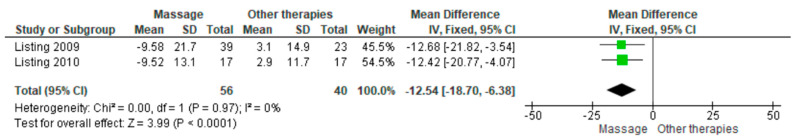
Forest plot of the posoled effect of massage therapy vs. placebo on Behavioral Symptoms Frequency (BSF) [23,24].

Massage therapy was compared with placebo in two studies evaluating the Behavioral Symptoms Frequency (BSF) scale. The pooled analysis showed a statistically significant reduction in symptom frequency in the massage group (MD = −12.54; 95% CI: −18.70 to −6.38; *p* < 0.0001) (Figure 4) [23,24]. The direction of effect was consistent across studies, and confidence intervals overlapped. No statistical heterogeneity was detected (I^2^ = 0%, *p* = 0.97). Visual inspection of the funnel plot suggested asymmetry, indicating potential publication bias. According to GRADE, the overall certainty of the evidence was rated as very low (Supplementary Table S4).

#### 3.4.2. Forest Plot QLQ Scale

**Figure 5:**
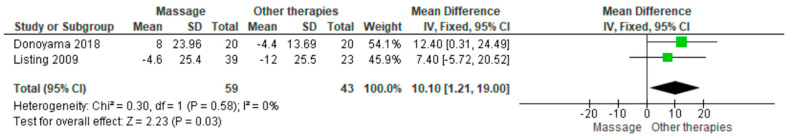
Forest plot of the pooled standardized mean difference for the EORTC Quality of Life Questionnaire (QLQ), comparing massage therapy with placebo [24,25].

Two studies compared massage therapy with placebo using the EORTC Quality of Life Questionnaire (QLQ). The pooled standardized mean difference demonstrated a statistically significant improvement favoring massage (SMD = 10.10; 95% CI: 1.21 to 19.00; *p* = 0.03) (Figure 5) [24,25]. Confidence intervals showed consistent direction of effect, and heterogeneity was low (I^2^ = 0%, *p* = 0.58). The certainty of evidence was rated as very low (Supplementary Table S4).

#### 3.4.3. Forest Plot HADS

**Figure 6:**
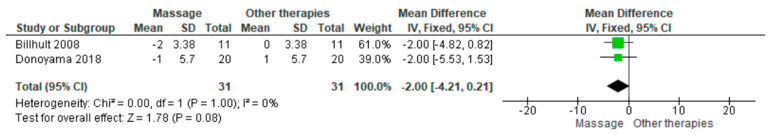
Forest plot showing the pooled mean difference for Hospital Anxiety and Depression Scale (HADS) scores in massage vs. placebo groups [6,25].

Two studies assessed anxiety and depression using the Hospital Anxiety and Depression Scale (HADS). The pooled analysis showed no statistically significant difference between massage and placebo (MD = −2.00; 95% CI: −4.21 to 0.21; *p* = 0.08) (Figure 6) [6,25]. Confidence intervals overlapped, and heterogeneity was absent (I^2^ = 0%, *p* = 1.00). The overall certainty of the evidence was rated as very low (Supplementary Table S4).

#### 3.4.4. Forest Plot STAI Scale

**Figure 7:**
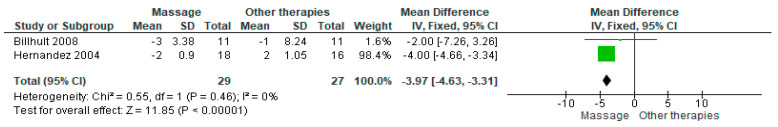
Forest plot of the pooled effect on state anxiety measured using the Spielberger State–Trait Anxiety Inventory (STAI) [6,7].

Two studies evaluated state anxiety using the Spielberger State–Trait Anxiety Inventory (STAI). Massage therapy produced a statistically significant reduction in anxiety scores compared with placebo (MD = −3.97; 95% CI: −4.63 to −3.31; *p* = 0.0001) (Figure 7) [6,7]. The direction of effect was consistent, and heterogeneity was low (I^2^ = 0%, *p* = 0.46). The certainty of evidence was rated very low (Supplementary Table S4).

#### 3.4.5. Forest Plot VAS

**Figure 8:**
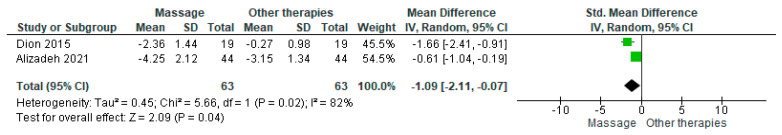
Forest plot showing the pooled mean difference in symptom intensity measured with the Visual Analog Scale (VAS) comparing massage therapy and placebo [18,27].

Two studies compared massage with placebo using the Visual Analog Scale (VAS) for symptom intensity. The pooled estimate showed a small but statistically significant benefit (MD = −1.09; 95% CI: −2.11 to −0.07; *p* = 0.04) (Figure 8) [5,18]. Substantial heterogeneity was detected (I^2^ = 82%, *p* = 0.02). The overall certainty of the evidence was judged as very low (Supplementary Table S4).

## 4. Discussion

Massage therapy encompasses a variety of manual techniques involving rhythmic compression, variable pressure, and structured movements aimed at producing relaxation and therapeutic benefits. Core techniques include friction, effleurage, petrissage, tapotement, shaking, and passive mobilization, all contributing to its mechanical and neurophysiological effects [27,30,31,32,33,34]. In this meta-analysis, massage therapy demonstrated improvements in selected clinical outcomes among cancer patients, although the overall certainty of the evidence was limited by methodological weaknesses within the included trials.

Findings from the current review partially align with those of prior systematic reviews and meta-analyses. Lee et al. (2015) evaluated 46 studies and reported significant reductions in pain-particularly postoperative pain- and also identified foot reflexology as more effective than body or aromatherapy massage [35]. Although our analysis did not focus on pain subtypes, we found a small but statistically significant reduction in symptom intensity on the VAS, supporting Lee et al.’s overall conclusions. Similarly, Pan et al. (2014) found that massage reduced anger and fatigue in breast cancer patients but produced no significant changes in depression, anxiety, pain, or quality of life [36]. Our findings are consistent with this pattern: the BSF scale demonstrated improvement, whereas HADS scores did not differ significantly between massage and control groups.

Zhang et al. (2023) reported beneficial effects of massage on cancer-related pain across multiple cancer types and suggested greater benefit for foot reflexology and manual acupressure [32]. Our results support this direction of effect, as the QLQ scale showed improved quality-of-life scores in patients receiving massage therapy. Similarly, Shan et al. (2023) found that massage and particularly reflexology was effective for cancer-related fatigue [23,25,37,38]. The improvement we observed on the BSF scale, which includes fatigue-related symptoms, aligns with Shan et al.’s conclusions.

Other systematic reviews have highlighted potential psychological benefits. Wilkinson et al. (2008) and Lee et al. (2011) reported short-term reductions in anxiety, although findings were inconsistent and limited by small sample sizes and lack of blinding [39,40]. In our analysis, a significant reduction in anxiety was observed using the STAI scale, while HADS showed no significant changes. This discrepancy likely reflects methodological differences between scales, the timing of assessments, or baseline symptom severity. Reviews by Shin et al. (2016) and Ernst (2009) also noted improvements in anxiety, pain, and fatigue, but emphasized the limited methodological quality of available studies, which is consistent with the low overall certainty in our findings [41,42].

Several physiological mechanisms may explain the benefits associated with massage therapy. Mechanically, massage enhances blood and lymphatic circulation, improving oxygen and nutrient delivery and facilitating the removal of metabolic waste products [27,30,31,32]. Mobilization of soft tissues stimulates proprioceptors, which increases neuromuscular relaxation through acetylcholine release. Endocrine modulation may also play a role: massage reduces cortisol and catecholamine secretion, promoting parasympathetic activation and generating decreases in heart and respiratory rates. These changes correspond with reductions in perceived anxiety and stress [33,34]. In breast cancer patients, massage has been associated with increased Natural Killer (NK) cell activity, possibly due to reduced cortisol levels [7]. The use of aromatherapy in some studies may further enhance relaxation through olfactory–limbic pathways [35].

The findings of the meta-analysis reinforce the idea that massage therapy may provide short-term relief for selected cancer-related symptoms. Improvements were observed in BSF, QLQ, STAI, and VAS outcomes, suggesting benefits in fatigue, behavioral symptoms, quality of life, anxiety, insomnia, and global symptom perception. However, the absence of significant effects in HADS implies that massage may exert stronger effects on momentary or state anxiety than on persistent psychological disorders.

Despite these favorable results, several clinical and methodological considerations must be acknowledged. All included studies used assessor-administered scales, and the lack of blinding may introduce detection bias. Massage outcomes are also sensitive to therapist characteristics, patient expectations, and the therapeutic environment, which could modify perceived symptom relief. Although adverse events were rare and generally mild, serious complications have been reported when massage is performed incorrectly or by untrained individuals [31].

This review has several limitations. First, considerable heterogeneity existed in cancer diagnoses, which may have diluted or exaggerated effect estimates. Second, most pooled outcomes were informed by only two studies, substantially limiting the reliability and generalizability of the findings. Third, methodological weaknesses particularly in blinding and allocation concealment lower confidence in the observed effects. Additionally, variation in massage modalities, session duration, and therapeutic frequency prevented meaningful subgroup analyses. Finally, visual inspection of funnel plots suggested potential publication bias. Overall, although massage therapy appears to offer short-term improvements in selected symptoms among cancer patients, the evidence remains constrained by low methodological quality and inconsistency across studies. Future trials should adopt standardized protocols, ensure adequate blinding where feasible, stratify analyses by cancer type, and include larger sample sizes to strengthen the robustness of conclusions.

## 5. Conclusions

Massage therapy appears to be a potentially beneficial complementary intervention for cancer patients, particularly for improving selected physical and psychological symptoms. In this meta-analysis, significant improvements were observed in fatigue, behavioral symptoms, quality-of-life domains, and overall symptom intensity, as reflected in the BSF, QLQ, and VAS scales. Although anxiety and depression outcomes measured through HADS and STAI did not consistently reach statistical significance, trends toward short-term emotional relief were noted, which are consistent with hypothesized physiological mechanisms such as reduced sympathetic activation.

Despite these findings, the overall certainty of the evidence remains very low due to methodological limitations, small sample sizes, heterogeneity in cancer types and intervention protocols, and risks of bias across included studies. Therefore, the observed benefits should be interpreted with caution. Massage therapy may still hold value within supportive and palliative care by providing relaxation, comfort, and non-pharmacological symptom relief. However, future research should prioritize rigorous trial designs, standardized massage protocols, clearly defined outcomes, and stratification by cancer type to better determine the clinical role of massage therapy in oncology settings.

## Figures and Tables

**Table 1:** Summary of studies on massage therapy in patients undergoing cancer treatment.

Author	Country	Population		Intervention		Outcomes	Follow-Up	Results
		Sample size (n)	Patients mean age (SD)	Intervention	Characteristics/Dose			
Mehling et al., 2007 [19]	USA	CG: 45 EG: 93	CG: 59.2 (1.7) EG: 5.,9 (1.9) Post-surgical cancer treatment	CG: Standard treatment EG: Acupuncture and massage	CG: Standard care; offered 30 min massage after completing POD3 questionnaire EG: Swedish massage + acupressure foot massage, 10–30 min/session (avg 20 ± 3 min)	NRS (nausea) Number of episodes of vomiting in the last 24 h. Likert scale (anxiety/tension) Likert scale (depression)	-	NRS (nausea) (*p* = 0.26) Number of episodes of vomiting in the last 24 h (*p* = 0.20) Likert scale (anxiety/tension) (*p* = 0.15) Likert scale (depression) (*p* = 0.003)
Billhult et al., 2008 [6]	Sweden	CG: 11 EG: 11	CG: 64 (9.1) EG: 61 (4.9) Radiation therapy for post-surgical breast cancer	CG: Standard treatment EG: Massage	CG: Equal attention, 20 min unstructured conversation (no massage) EG: Effleurage massage, 20 min × 10 sessions over 3–4 weeks; feet/lower legs or hands/lower arms; standardized light pressure	HADS-D (depression) HADS-A (anxiety) LSQ (quality of life) S-STAI (state anxiety) T-STAI (trait anxiety) Blood samples (NK Cell Cytotoxicity)	-	HADS-D (depression) (*p* = 0.382) HADS-A (anxiety) (*p* = 0.152) LSQ (quality of life) (*p* = 0.032) S-STAI (state anxiety) (*p* = 0.091) T-STAI (trait anxiety) (*p* = 0.087) Blood samples (NKCC) (*p* = 0.025)
Dion et al., 2015 [18]	USA	CG: 19 EG: 19	CG (group 2): 47.37 (9.24) EG (group 1): 47.95 (7.71) Post-surgical breast cancer patients	CG: Standard treatment EG: Massage	CG: 15 min paced breathing DVD + gratitude meditation + 20-min individualized massage instruction; encouraged post-discharge practice EG: 20-min massage × 3 days by licensed therapist; individualized (techniques, pressure, areas, positioning); Swedish massage, acupressure and foot reflexology	VAS (stress/anxiety/relaxation/insomnia/alertness/fatigue/tension/pain/mood/energy) PSS-14 (stress)	Day 1, day 2, day 3 and 3-week follow-up phone call	VAS (stress/anxiety/relaxation/insomnia/alertness/fatigue/tension/pain/mood/energy) (*p* = 0.089) PSS-14 (stress) (*p* = 0.090)
Kutner et al., 2008 [20]	USA	CG: 192 EG: 188	CG: 64.2 (14.4) EG: 65.2 (14.4) Advanced cancer with moderate to severe pain	CG: Standard treatment EG: Massage	CG: Simple touch control, 3 min light hand contact on multiple sites; matched time/attention; no bodywork experience. EG: Gentle effleurage 65%/35% + trigger point release; adapted for fragile skin, edema, positions supine, seated side lying; provided by licensed therapists	Mood (MPAC) Pain (MPAC) Mean pain (BPI) Worst pain (BPI) Pain interference (BPI) Global distress (MSAS) Physical symptoms (MSAS) Psychological symptoms (MSAS) Quality of life (MQOL) Physical Well-Being (MQOL) Existential (MQOL) Support (MQOL) Heart rate Respiratory rate Morphine Parenteral Use	Face-to-face interviewer-administered questionnaires, over 3 weeks.	Mood (MPAC) (*p* < 0.0001) Pain (MPAC) (*p* < 0.0001) Mean pain (BPI) (*p* = 0.66) Worst pain (BPI) (*p* = 0.53) Pain interference (BPI) (*p* = 0.60) Global distress (MSAS) (*p* = 0.97) Physical symptoms (MSAS) (*p* = 0.62) Psychological symptoms (MSAS) (*p* = 0.50) Quality of life (MQOL) (*p* = 0.73) Physical Well-Being (MQOL) (*p* = 0.51) Existential (MQOL) (*p* = 0.53) Support (MQOL) (*p* = 0.36) Heart rate (*p* = 0.08) Respiratory rate (*p* = 0.16) Morphine Parenteral (*p* = 0.17)
Lopez et al., 2022 [21]	USA	CG (Group3): 8 CG (Group4): 11 EG (Group1): 16 EG (Group 2): 18	CG + EG (all participants, not reported by group): 60.8 (9.8) Chemotherapy -induced peripheral neuropathy CIPN	CG (groups 3 and 4): Alternative site massages EG (groups 1 and 2): Massage followed by a CIPN protocol	CG: Alternate site massage (scalp, neck, shoulders, upper back); adjusted for lymphedema/health conditions; session frequency matched to EG (2–3 times per week). EG: Site-specific lower extremity massage ≤30 min per session; semi-Fowler position; hypoallergenic lotion; Group 1: 3 times/week for 4 weeks, Group 2: 2 times/week for 6 weeks; Both groups followed a standardized CIPN protocol	PQAS-DP (deep pain) PQAS-PP (paroxysmal pain) PQAS-SP (Surface pain)	10-week follow-up	PQAS-DP (deep pain) (*p* = 0.453) PQAS-PP (paroxysmal pain) (*p* = 0.260) PQAS-SP (surface pain) (*p* = 0.108)
Toth et al., 2013 [22]	USA	CG: 10 EG: 20	CG: 54.9 EG: 54.9 Home-based cancer treatment in patients with metastatic cancer	CG: Standard “non-curative” treatment EG: Swedish and non-Swedish therapeutic massages	CG: No-touch control; therapists remained near patients for 15–45 min without physical contact EG: Massage 15–45 min; Swedish & non-Swedish techniques (effleurage, petrissage, compression, stretching, rocking, myofascial release, range-of-motion (ROM) exercises, acupressure, craniosacral); aimed at promoting calming and centering effects	Pain anxiety alertness physical well-being psychological well-being McGill total sleep Data reported as median (Q1, Q3)	1-month follow-up	Pain (*p* = 0.65/0.14) anxiety (*p* = 0.92/0.85) alertness ( *p*= 0.10/0.44) physical well-being (*p* = 0.005/0.27) psychological well-being (*p* = 0.15/0.23) McGill total (*p* = 0.03/0.33) sleep (*p* = 0.25/0.49)
Listing et al., 2010 [23]	Germany	CG: 17 EG: 17	CG: 59.9 (11.5) GE: 59.5 (12.1) Cancer treatment in women with primary breast cancer	CG: Standard treatment *EG: Swedish* therapeutic Massage	*CG:* No additional therapy for 11 weeks; instructed to begin Jacobson’s progressive muscle relaxation after study completion EG: Swedish massage 30 min; prone position; stroking, kneading, friction techniques	Tension (PSQ) Demands (PSQ) Worries (PSQ) Joy (PSQ) Anger (PSQ) Anxious depression (BSF) Listlessness (BSF) Tiredness (BSF) Elevated Mood (BSF) Involvement (BSF)	6-week follow-up	Tension (PSQ) (*p* = 0.52) Demands (PSQ) (*p* = 0.25) Worries (PSQ) (*p* = 0.12) Joy (PSQ) (*p* = 0.07) Anger (PSQ) (*p* = 0.82) Anxious depression (BSF) (*p* = 0.90) Listlessness (BSF) (*p* = 0.28) Tiredness (BSF) (*p* = 0.79) Elevated mood (BSF) (*p* = 0.55) Involvement (BSF) (*p* = 0.57)
Listing et al., 2009 [24]	Germany	CG: 23 EG: 39	CG: 61.4 (10.9) EG: 57.6 (10.8) Cancer treatment in women with breast cancer	CG: Standard treatment EG: Swedish Massage	CG: No additional therapy during 11-week study EG: Swedish massage 30 min × 2/week; back, neck, head; pressure tailored to patient comfort	Body Pain (SF-8) Pain of Limbs (GBB) Fatigue (GBB) Breast Symptoms (QLQ-BR23) Arm Symptoms (QLQ-BR23) Tiredness (BSF) Anger (BSF) Anxious Depression (BSF) Listlessness (BSF) Involvement (BSF) Elevated Mood (BSF)	11-week follow-up	Body pain (SF-8) (*p* = 0.06) Pain of limbs (GBB) (*p* = 0.49) Fatigue (GBB) (*p* = 0.45) Breast symptoms (QLQ-BR 23) (*p* = 0.02) Arm symptoms (QLQ-BR23) (*p* = 0.48) Tiredness (BSF) (*p* = 0.03) Anger (BSF) (*p* = 0.02) Anxious depression (BSF) (*p* = 0.007) Listlessness (BSF) (*p* = 0.48) Involvement (BSF) (*p* = 0.68) Elevated mood (BSF) (*p* = 0.016)
Hernandez-Reif et al., 2004 [7]	USA	CG: 16 EG: 18	CG: 53.3 (11.7) GE: 52.7 (9.5) Treatment in women with postoperative breast cancer	CG: Standard Treatment EG: Massage of head, arms, legs/feet, and back)	CG: Standard treatment; offered massage after 5-week study EG: Massage 30 min × 3/week for 5 weeks; head, arms, legs/feet, back; stroking, squeezing, stretching	Symptom Checklist (SCL-90-R): Anxiety Hostility Depression (SCL-90-R) and Depression (POMS) Anger (POMS) Anxiety (STAI) Urinary biochemistry Creatinine Cortisol Norepinephrine Epinephrine Dopamine Serotonin Immune measures NK cell numbers (NKCC) Lymphocytes	5-week study period.	Symptom checklist (SCL-90-R): Depression (*p* = 0.01) Hostility (*p* = 0.05) Depression POMS (*p* = 0.05) Anger POMS (*p* = 0.05) Anxiety STAI (*p* = 0.05) Urinary biochemistry Creatinine Cortisol Norepinephrine (*p* = 0.05) Epinephrine Dopamine (*p* = 0.05) Serotonin levels (*p* = 0.05) Immune measures NK cell numbers (did not attain significance) (*p* => 0.05) NK Cell Cytotoxicity (*p* => 0.05) Lymphocytes (*p* = 0.05)
Donoyama et al., 2018 [25]	Japan	CG: 20 EG: 20	CG: 55.5 EG: 53: Survivors of various gynecologic cancers	CG: Standard treatment EG: Anma-massage	*CG:* 40-min relaxing conversation; received a single Anma session after 2 months (outside study intervention) *EG:* Anma massage 40 min × 8 weeks; kneading, stroking, pressing; delivered by the same therapist	- HADS Anxiety (Japan version) - HADS Depression (Japan version) - EORTC QLQ-C30 symptom scales: Fatigue Nausea and vomiting Pain Dyspnea Insomnia Appetite loss Constipation Diarrhea Financial difficulties - EORTC QLQ-C30 Global and functioning scales: Global Health Status/QOL Physical Functioning Role Functioning Emotional Functioning Cognitive Functioning Social Functioning	Intervention period: 8 weeks.	- HADS Anxiety (Japan version) (*p* = 0.256) - HADS Depression (Japan version) (*p* = 0.282) - EORTC QLQ-C30 symptom scales: Fatigue (*p* = 0.047) Nausea and vomiting (*p* = 0.506) Pain (*p* = 0.682) Dyspnea (*p* = 0.277) Insomnia (*p* = 0.003) Appetite loss (*p* = 0.226) Constipation (*p* = 0.158) Diarrhea (*p* = 1.000) Financial difficulties (*p* = 1.000) - EORTC QLQ-C30 Global and functioning scales: Global Health Status/QOL (*p* = 0.042) Physical Functioning (*p* = 0.755) Role Functioning (*p* = 0.919) Emotional Functioning (*p* = 0.895) Cognitive Functioning (*p* = 0.618) Social Functioning (*p* = 0.626)
Campeau et al., 2007 [26]	Canada	CG: 48 EG: 52	CG: 58 EG: 60 Patients undergoing radiotherapy for cancer	CG: No massage EG: Massage of various areas of the body (feet, back, arms, hands, neck, and scalp)	*CG:* No massage; therapist met participant before radiation to assess anxiety EG: Chair massage 15 min × 10 sessions; effleurage & petrissage; back, arms, hands, neck, scalp; avoided irradiated areas	VAS (pain) VAS-anxiety	6 months follow-up (January 2006–June 2006)	VAS-anxiety (*p* = 0.73). VAS Pain (*p* = 0.03)
Alizadeh et al., 2021 [27]	Iran	CG: 44 EG: 44	CG: 31.5 EG: 42.5 Cancer treatment in patients with gastric cancer after chemotherapy		CG: Same procedures as EG; no foot massage during chemotherapy; communication and interaction time matched EG: Foot massage 14 min × 4 per chemo cycle (56 min total); superficial & deep effleurage, petrissage, friction; gentle pressure	VAS-F (fatigue)	Immediately post-chemotherapy and 24 h later	VAS-F (fatigue) (*p* = 0.001)

SD: Standard deviation CG: Control group; EG: Experimental group; VAS: Visual Analogue Scale; SF-8: TM Short Form-8 Health Survey TM; GBB: Giessen Complaints Inventory; STAI: State Trait Anxiety Inventory; HADS-A: Hospital Anxiety and Depression Scale—Anxiety; HADS-D: Hospital Anxiety and Depression Scale—Depression; SCL-90-R: Symptom Checklist-90-R; LSQ: Life Satisfaction Questionnaire; FACIT-F: Functional Assessment of Chronic Illness Therapy-Fatigue; FACT-B:D Functional Assessment of Cancer Therapy; TOI: Trial outcome index; SF-36: Health-related quality of life 36; PFS: Piper Fatigue Scale; BFI: Brief Fatigue Inventory; POMS: Profile of Mood State Questionnaire; BSF: Berlin Mood Questionnaire; MRS: Mood Rating Scale; ULL27: Upper Limb Lymphedema Questionnaire; EORTC QLQ-BR23: European Organisation for Research and Treatment of Cancer Quality of Life Questionnaire—breast cancer 23; PPT: Pressure Pain Thresholds.

## Data Availability

No new data were created or analyzed in this study.

## Supplementary Materials

The following supporting information can be downloaded at: https://www.mdpi.com/article/10.3390/healthcare13243268/s1, Supplementary Table S1: Details of the search strategy. Supplementary Table S2: Excluded studies and the reasons for their exclusion. Supplementary Table S3: PRISMA checklist. Supplementary Table S4: GRADE.
