# Supplementary material for: Efficacy of Massage Therapy for Symptom Management in Cancer Patients: A Systematic Review and Meta-Analysis
Source: Healthcare (Basel). 2025 Dec 12;13(24):3268. doi: 10.3390/healthcare13243268 (PMC12732605; doi:10.3390/healthcare13243268)
Supplement: Supplementary file 1 [file healthcare-13-03268-s001.zip › healthcare-3788521-supplementary.pdf]

Supplementary Tables

Supplementary Table S1: Details of the search *strategy.*

Database	*Search strategy*	*Results*	
		20-02-25	***21-05-2025***
Medline	***((("massage"[MeSH Terms] OR "massage"[All Fields] OR ("massage"[All Fields] AND "therapy"[All Fields]) OR "massage therapy"[All Fields] OR (("musculoskeletal manipulations"[MeSH Terms] OR ("musculoskeletal"[All Fields] AND "manipulations"[All Fields]) OR "musculoskeletal manipulations"[All Fields] OR "reflexology"[All Fields]) AND ("therapeutics"[MeSH Terms] OR "therapeutics"[All Fields] OR "therapies"[All Fields] OR "therapy"[MeSH Subheading] OR "therapy"[All Fields] OR "therapy s"[All Fields] OR "therapys"[All Fields])) OR (("massage"[MeSH Terms] OR "massage"[All Fields] OR "massages"[All Fields] OR "massaged"[All Fields] OR "massager"[All Fields] OR "massagers"[All Fields] OR "massaging"[All Fields]) AND ("musculoskeletal manipulations"[MeSH Terms] OR ("musculoskeletal"[All Fields] AND "manipulations"[All Fields]) OR "musculoskeletal manipulations"[All Fields] OR "reflexology"[All Fields]))) AND ("cancer s"[All Fields] OR "cancerated"[All Fields] OR "canceration"[All Fields] OR "cancerization"[All Fields] OR "cancerized"[All Fields] OR "cancerous"[All Fields] OR "neoplasms"[MeSH Terms] OR "neoplasms"[All Fields] OR "cancer"[All Fields] OR "cancers"[All Fields])) NOT ("review"[Publication Type] OR "review literature as topic"[MeSH Terms] OR "review"[All Fields])) AND ((clinicaltrial[Filter] OR randomizedcontrolledtrial[Filter]) AND (humans[Filter]))***	***323***	***326***
Wos	***massage therapy OR reflexology therapy OR massage reflexology AND cancer NOT review Filters: Clinical Trial, Randomized Controlled Trial, Humans***	***49***	***49***
CINAHL	***mediterranean diet AND metabolic syndrome Filters: Clinical Trial, Randomized Controlled Trial***	***10***	***10***
SCOPUS	***mediterranean diet AND metabolic syndrome Filters: Clinical Trial, Randomized Controlled Trial***	***33***	***33***
Google scholar	***mediterranean diet AND metabolic syndrome Filters: Clinical Trial, Randomized Controlled Trial***	***101***	***102***
Embase	***mediterranean diet AND metabolic syndrome Filters: Clinical Trial, Randomized Controlled Trial***	***22***	***23***
	Total	***548***	***553***

**** All searches were carried out on May 21, 2025.***

Supplementary Table S2: Excluded studies and the reasons for their exclusion.

N°	Reference	Reason
1	Bouhassira D, Wilhelm S, Schacht A, Perrot S, Kosek E, Cruccu G, Freynhagen R, Tesfaye S, Lledó A, Choy E, Marchettini P, Micó JA, Spaeth M, Skljarevski V, Tölle T. Neuropathic pain phenotyping as a predictor of treatment response in painful diabetic neuropathy: data from the randomized, double-blind, COMBO-DN study. Pain. 2014 Oct;155(10):2171-9. doi: 10.1016/j.pain.2014.08.020. Epub 2014 Aug 27. PMID: 25168665.	Mixes duloxetine with a variety of antidepressants in different doses.
2	Boyle J, Eriksson ME, Gribble L, Gouni R, Johnsen S, Coppini DV, Kerr D. Randomized, placebo-controlled comparison of amitriptyline, duloxetine, and pregabalin in patients with chronic diabetic peripheral neuropathic pain: impact on pain, polysomnographic sleep, daytime functioning, and quality of life. Diabetes Care. 2012 Dec;35(12):2451-8. doi: 10.2337/dc12-0656. Epub 2012 Sep 18. PMID: 22991449; PMCID: PMC3507552.	Mixes duloxetine with a variety of antidepressants in different doses.
3	Gaynor PJ, Liu P, Weller MA, Wohlreich MM. Comparison of safety outcomes among Caucasian, Hispanic, Black, and Asian patients in duloxetine studies of chronic painful conditions. Curr Med Res Opin. 2013 May;29(5):549-60. doi: 10.1185/03007995.2013.784191. Epub 2013 Apr 3. PMID: 23477538.	Evaluates adverse reactions to medications in different races.
4	Irving G, Tanenberg RJ, Raskin J, Risser RC, Malcolm S. Comparative safety and tolerability of duloxetine vs. pregabalin vs. duloxetine plus gabapentin in patients with diabetic peripheral neuropathic pain. Int J Clin Pract. 2014 Sep;68(9):1130-40. doi: 10.1111/ijcp.12452. Epub 2014 May 18. PMID: 24837444.	Evaluates adverse reactions to pregabalin, duloxetine, and both together.
5	Kaur H, Hota D, Bhansali A, Dutta P, Bansal D, Chakrabarti A. A comparative evaluation of amitriptyline and duloxetine in painful diabetic neuropathy: a randomized, double-blind, cross-over clinical trial. Diabetes Care. 2011 Apr;34(4):818-22. doi: 10.2337/dc10-1793. Epub 2011 Feb 25. PMID: 21355098; PMCID: PMC3064034.	Mixes duloxetine with a variety of antidepressants in different doses.
6	Marchettini P, Wilhelm S, Petto H, Tesfaye S, Tölle T, Bouhassira D, Freynhagen R, Cruccu G, Lledó A, Choy E, Kosek E, Micó JA, Späth M, Skljarevski V, Lenox-Smith A, Perrot S. Are there different predictors of analgesic response between antidepressants and anticonvulsants in painful diabetic neuropathy? Eur J Pain. 2016 Mar;20(3):472-82. doi: 10.1002/ejp.763. Epub 2015 Aug 27. PMID: 26311228.	Mixes duloxetine with a variety of antidepressants in different doses.
7	Raskin J, Wang F, Pritchett YL, Goldstein DJ. Duloxetine for patients with diabetic peripheral neuropathic pain: a 6-month open-label safety study. Pain Med. 2006 Sep-Oct;7(5):373-85. doi: 10.1111/j.1526-4637.2006.00207.x. PMID: 17014595.	Compares same dose of duloxetine in different modalities.
8	Rizea-Savu S, Duna SN, Ghita A, Iordachescu A, Chirila M. The Effect of Food on the Single-Dose Bioavailability and Tolerability of the Highest Marketed Strength of	Evaluates bioavailability of

	Duloxetine. Clin Pharmacol Drug Dev. 2020 Oct;9(7):797-804. doi: 10.1002/cpdd.759. Epub 2019 Dec 2. PMID: 31793229; PMCID: PMC7586977.	duloxetine in association with food.
9	Skljarevski V, Desaiah D, Zhang Q, Chappell AS, Detke MJ, Gross JL, Ziegler D. Evaluating the maintenance of effect of duloxetine in patients with diabetic peripheral neuropathic pain. Diabetes Metab Res Rev. 2009 Oct;25(7):623-31. doi: 10.1002/dmrr.1000. PMID: 19637208.	Mixes different doses of duloxetine with each other.
10	Skljarevski V, Desaiah D, Liu-Seifert H, Zhang Q, Chappell AS, Detke MJ, Iyengar S, Atkinson JH, Backonja M. Efficacy and safety of duloxetine in patients with chronic low back pain. Spine (Phila Pa 1976). 2010 Jun 1;35(13):E578-85. doi: 10.1097/BRS.0b013e3181d3cef6. PMID: 20461028.	Evaluates chronic low back pain.
11	Tanenberg RJ, Irving GA, Risser RC, Ahl J, Robinson MJ, Skljarevski V, Malcolm SK. Duloxetine, pregabalin, and duloxetine plus gabapentin for diabetic peripheral neuropathic pain management in patients with inadequate pain response to gabapentin: an open-label, randomized, non-inferiority comparison. Mayo Clin Proc. 2011 Jul;86(7):615-26. doi: 10.4065/mcp.2010.0681. PMID: 21719618; PMCID: PMC3127557.	Mixes duloxetine with a variety of antidepressants in different doses.
12	Tanenberg RJ, Clemow DB, Giaconia JM, Risser RC. Duloxetine Compared with Pregabalin for Diabetic Peripheral Neuropathic Pain Management in Patients with Suboptimal Pain Response to Gabapentin and Treated with or without Antidepressants: A Post Hoc Analysis. Pain Pract. 2014 Sep;14(7):640-8. doi: 10.1111/papr.12121. Epub 2013 Oct 24. PMID: 24152185.	Mixes duloxetine with a variety of antidepressants in different doses.
13	Tesfaye S, Wilhelm S, Lledo A, Schacht A, Tölle T, Bouhassira D, Cruccu G, Skljarevski V, Freynhagen R. Duloxetine and pregabalin: high-dose monotherapy or their combination? The "COMBO-DN study"--a multinational, randomized, double-blind, parallel-group study in patients with diabetic peripheral neuropathic pain. Pain. 2013 Dec;154(12):2616-2625. doi: 10.1016/j.pain.2013.05.043. Epub 2013 May 31. PMID: 23732189.	Mixes duloxetine with a variety of antidepressants in different doses.
14	Wernicke JF, Prakash A, Kajdasz DK, Houston J. Safety and tolerability of duloxetine treatment of diabetic peripheral neuropathic pain between patients with and without cardiovascular conditions. J Diabetes Complications. 2009 Sep-Oct;23(5):349-59. doi: 10.1016/j.jdiacomp.2008.07.004. Epub 2008 Sep 2. PMID: 18768332.	Evaluates safety in patients with cardiovascular diseases.
15	Wu EQ, Birnbaum HG, Mareva MN, Le TK, Robinson RL, Rosen A, Gelwicks S. Cost-effectiveness of duloxetine versus routine treatment for U.S. patients with diabetic peripheral neuropathic pain. J Pain. 2006 Jun;7(6):399-407. doi: 10.1016/j.jpain.2006.01.443. PMID: 16750796.	Evaluates the monetary cost-benefit of duloxetine treatment.
16	Yarnitsky D, Granot M, Nahman-Averbuch H, Khamaisi M, Granovsky Y. Conditioned pain modulation predicts duloxetine efficacy in painful diabetic neuropathy. Pain. 2012 Jun;153(6):1193-1198. doi: 10.1016/j.pain.2012.02.021. Epub 2012 Apr 3. PMID: 22480803.	Mixes different doses of duloxetine with each other.
17	Yasuda H, Hotta N, Kasuga M, Kashiwagi A, Kawamori R, Yamada T, Baba Y, Alev L, Nakajo K. Efficacy and safety of 40 mg or 60 mg duloxetine in Japanese adults with diabetic neuropathic pain: Results from a randomized, 52-week, open-label study. J Diabetes Investig. 2016 Jan;7(1):100-8. doi: 10.1111/jdi.12361. Epub 2015 May	Mixes the results of groups of 40 and 60 mg/d of duloxetine.

	18. PMID: 26816607; PMCID: PMC4718094.	
18	Yuen E, Gueorguieva I, Bueno-Burgos L, Iyengar S, Aarons L. Population pharmacokinetic/pharmacodynamic models for duloxetine in the treatment of diabetic peripheral neuropathic pain. Eur J Pain. 2013 Mar;17(3):382-93. doi: 10.1002/j.1532-2149.2012.00209.x. Epub 2012 Aug 14. PMID: 22893563.	Evaluates PK/PD pharmacokinetic model but not pain.

Supplementary Table 3: PRISMA checklist

Section and Topic	Item #	Checklist item
**TITLE**		
Title	1	Identify the report as a systematic review.
**ABSTRACT**		
Abstract	2	See the PRISMA 2020 for Abstracts checklist.
**INTRODUCTION**		
Rationale	3	Describe the rationale for the review in the context of existing knowledge.
Objectives	4	Provide an explicit statement of the objective(s) or question(s) the review addresses.
**METHODS**		
Eligibility criteria	5	Specify the inclusion and exclusion criteria for the review and how studies were grouped for the synt
Information sources	6	Specify all databases, registers, websites, organisations, reference lists and other sources searched or co Specify the date when each source was last searched or consulted.
Search strategy	7	Present the full search strategies for all databases, registers and websites, including any filters and lir
Selection process	8	Specify the methods used to decide whether a study met the inclusion criteria of the review, inclu screened each record and each report retrieved, whether they worked independently, and if applica tools used in the process.
Data collection process	9	Specify the methods used to collect data from reports, including how many reviewers collected data they worked independently, any processes for obtaining or confirming data from study investigator of automation tools used in the process.
Data items	10a	List and define all outcomes for which data were sought. Specify whether all results that were com domain in each study were sought (e.g.,, for all measures, time points, analyses), and if not, the met results to collect.
	10b	List and define all other variables for which data were sought (e.g., participant and intervention chara Describe any assumptions made about any missing or unclear information.
Study risk of bias assessment	11	Specify the methods used to assess risk of bias in the included studies, including details of the tool(s) assessed each study and whether they worked independently, and if applicable, details of automatio

Section and Topic	Item #	Checklist item
Effect measures	12	Specify for each outcome the effect measure(s) (e.g., risk ratio, mean difference) used in the synthesis
Synthesis methods	13a	Describe the processes used to decide which studies were eligible for each synthesis (e.g., tabulat characteristics and comparing against the planned groups for each synthesis (item #5)).
	13b	Describe any methods required to prepare the data for presentation or synthesis, such as handling of r or data conversions.
	13c	Describe any methods used to tabulate or visually display results of individual studies and syntheses
	13d	Describe any methods used to synthesize results and provide a rationale for the choice(s). If met describe the model(s), method(s) to identify the presence and extent of statistical heterogeneity, and s
	13e	Describe any methods used to explore possible causes of heterogeneity among study results (e.g., regression).
	13f	Describe any sensitivity analyses conducted to assess robustness of the synthesized results.
Reporting bias assessment	14	Describe any methods used to assess risk of bias due to missing results in a synthesis (arising from re
Certainty assessment	15	Describe any methods used to assess certainty (or confidence) in the body of evidence for an outcome
**RESULTS**		
Study selection	16a	Describe the results of the search and selection process, from the number of records identified in th studies included in the review, ideally using a flow diagram.
	16b	Cite studies that might appear to meet the inclusion criteria, but which were excluded, and explain w
Study characteristics	17	Cite each included study and present its characteristics.
Risk of bias in studies	18	Present assessments of risk of bias for each included study.
Results of individual studies	19	For all outcomes, present, for each study: (a) summary statistics for each group (where appropriate) and its precision (e.g., confidence/credible interval), ideally using structured tables or plots.
Results of syntheses	20a	For each synthesis, briefly summarise the characteristics and risk of bias among contributing studies.
	20b	Present results of all statistical syntheses conducted. If meta-analysis was done, present for each the precision (e.g. confidence/credible interval) and measures of statistical heterogeneity. If comparing gro of the effect.
	20c	Present results of all investigations of possible causes of heterogeneity among study results.
	20d	Present results of all sensitivity analyses conducted to assess the robustness of the synthesized results
Reporting biases	21	Present assessments of risk of bias due to missing results (arising from reporting biases) for each synt
Certainty of evidence	22	Present assessments of certainty (or confidence) in the body of evidence for each outcome assessed.
**DISCUSSION**		
Discussion	23a	Provide a general interpretation of the results in the context of other evidence.
	23b	Discuss any limitations of the evidence included in the review.
	23c	Discuss any limitations of the review processes used.
	23d	Discuss implications of the results for practice, policy, and future research.
**OTHER INFORMATION**		
Registration and protocol	24a	Provide registration information for the review, including register name and registration number, o not registered.
	24b	Indicate where the review protocol can be accessed, or state that a protocol was not prepared.

Section and Topic	Item #	Checklist item
	24c	Describe and explain any amendments to information provided at registration or in the protocol.
Support	25	Describe sources of financial or non-financial support for the review, and the role of the funders or sp
Competing interests	26	Declare any competing interests of review authors.
Availability of data, code and other materials	27	Report which of the following are publicly available and where they can be found: template data colle from included studies; data used for all analyses; analytic code; any other materials used in the revie

From: Page MJ, McKenzie JE, Bossuyt PM, Boutron I, Hoffmann TC, Mulrow CD, et al. The PRISMA 2020 statement: an updated guideline for reporting systematic reviews. BMJ 2021;372:n71. doi: 10.1136/bmj.n71

For more information, visit: http://www.prisma-statement.org/

Supplementary Table 4: GRADE

Supplemental Digital Content. Table S3. Summary of Findings (SoF) and quality of evidence (GRADE) for Acupuncture treatment in patients with diabetes mellitUS.

Certainty assessment							№ of patients		Effect		Quality of evidence (GRADE)	Importance
№ of studies	Study design	Risk of bias	Inconsistency	Indirectness	Imprecision	Other considerations	Acupuncture	Control group	Relative (95% CI)	SMD (95% CI)		
BSF scale												
**2**	RCT	Not Serious	Serious	Not serious	Serious	None	56	40	-	-12.54 (-18.70 to 6.38)	⊕⊕◯◯ Low	CRITICAL
QLQ scale												
2	RCT	Not Serious	Very serious	Not serious	Serious	None	59	43	-	10.10 (1.21 to 19.00)	⊕◯◯◯ Very low	CRITICAL
HADS scale												
2	RCT	Not serious	Very serious	Not serious	Serious	None	31	31	-	-2.00 (-4.21 to 0.21)	⊕⊕◯◯ Low	CRITICAL

STAI scale

2	RCT	Not Serious	Very serious	Not serious	Serious	None	29	27	-	-3.97 (-4.63 to -3.31)	⊕⊕○○ Low	IMPORTANT

VAS scale

2	RCT	Not serious	Serious	Not serious	Serious	None	63	63	-	-1.09, (-2.11 to 0.07)	⊕⊕○○ Low	IMPORTANT

SMD: Standard Mean Difference; **RCT:** Randomized clinical trial; **Quality of evidence:** High: The research provides a very good indication of the likely effect. The probability that the effect is different is low; Moderate: The research provides a good indication of the likely effect. The probability that the effect is substantially different is moderate. Low: The research gives some indication of the probable effect. However, the probability that the effect is substantially different is high. Very low: The research does not provide a reliable estimate of the probable effect. The probability that the effect is substantially different is very high. **Downgrading:** GRADE approach has four reasons for possible rate down the quality of evidence. Begins with the study designs (trials or observational studies), secondly downgrading the evidence one level: (1) for study limitation, if the majority of studies (>50%) was rated as high risk of bias; (2) for inconsistency, if heterogeneity was greater than the accepted low level (I^2 >40%); (3) for indirectness, directness was undoubled; (4) for imprecision, if meta-analysis had a small sample size (n <400) or confidence interval was very wide.
